# A personal reflection: What I learned about diversity, inclusivity and equity because of a young homeless man and his dog

**DOI:** 10.1177/13591045251326704

**Published:** 2025-03-04

**Authors:** Pierre-Paul Tellier

**Affiliations:** Department of Family Medicine, McGill University, Canada

**Keywords:** Homelessness, adolescent and young adult, youth homelessness, diversity, inclusivity, equity

## Abstract

Who are homeless youths? This is the question I asked after observing a young man and his dog in the lobby of a bank on a cold January evening in Montreal. In attempting to answer this question I found that a universally accepted definition for “homeless youth” does not exist. Nonetheless, research exist that define who they are, the issues associated with homelessness and the health risks they face on the street. This led me wo ask what I could have done to help the young man. My experience help me realize that the street in unfortunately not only diverse, but also potentially inclusive of everyone, but like the rest of society it is not necessarily equitable.

## Plain Language Summary

Given the tone of this article the abstract I believe is in plain language and summarizes the article.

For this special edition on Diversity, Equity and Inclusion I have chosen to tell you a story which starts at 18:00 hours on a frigid cold weeknight in January, in Montreal. I was in clinic and had just finished making phone calls to patients to discuss the results of tests that had been ordered for them. The clinic is on the first floor of the building, so I went down to the ground floor to order and wait for an UBER, to go home. This space is shared with a pharmacy, that was closed and a bank. Though the bank was also closed, the lobby is always accessible to clients and can be seen from where I sat. I could see that a young white male, about 16–17 years of age, was sitting there, on the ground, huddled with a dog. He had a backpack and at one point he pulled out a blanket and wrapped it around his dog. I watched as people entered, to access the ATM, clearly avoiding him.

My car arrived, I got up and left. However, as I sat in the warm vehicle going home, I felt guilty about leaving without acknowledging the young person and his dog, and I started wondering who is this youth and why is he there? I assumed that he was a homeless person seeking to protect himself from the cold on this wintry evening. However, this was an assumption, what did I really know about him? What could I have done to help him and his dog, his only companion.

## Defining youth homelessness

Over the years I have worked in a variety of clinical settings encountering and talking to some homeless youth, hence I was aware of some of the issues associated with homelessness and the health risk they face on the street, but at this point I needed to know more. Research has been done with this population, but despite the research a universally accepted definition for “youth homelessness” does not exist.

Since there is no single definition for youth homelessness why not examine the components of the expression. The United Nations define youth: “The UN Secretariat uses the term youth and young people interchangeable to mean age 15–24 with the understanding that member states and other entities use different definitions” (United Nations, n.d). They freely admit that there is no universally accepted definition of this term, that the meaning of youth varies throughout the world, and they use theirs to guide statistical analysis (United Nations, n.d). However, there is more to understanding adolescents and young adults (AYA) than an age range. On the same page where this cursory definition is presented the UN does provide a lead as to what this portion of the life cycle is about. The writers state “YOUTH is best understood as a period of transition from the dependence of childhood to adulthood independence” (United Nations, n.d). In their article entitled “The ABC of adolescence – Adolescent development” Christie and Viner outline the developmental tasks of this group. These include biological and sexual maturation, personal identity formation, the development of intimate sexual relationships with an appropriate peer and establishment of independence and autonomy in the context of the sociocultural environment (Christie & Viner, 2005). Therefore, in the case of homeless youth, we must consider the impact of the sociocultural environment, the street with its associated risks, in which they find themselves to complete these developmental tasks.

Let us now consider “homelessness”. If we again turn ourselves to the United Nations, we find the definition in a document presented by the Special Rapporteur, Ms Leilani Farah, on the right to adequate housing to the Human Rights Council in 2020, “Guidelines for the implementation of the Rights to Adequate Housing”. In these guidelines we find this definition: “Homelessness is a profound assault on dignity, social inclusion and the right to life. It is a prima facie violation of the right to housing and violates a number of other human rights in addition to the right to life, including non-discrimination, health, water and sanitation, security of the person and freedom from cruel, degrading and inhuman treatment” (United Nations Human Rights Council, 1989). This speaks to the harsh reality that young persons who are living on the street face daily, at a critical time in their life. One then wonders how does lack of proper nutrition and water affect physical growth, how does the stress, lack of security, and “degrading and inhuman treatment” impact on their emotional and psychological development? This leads one to realize that the answer to who is this young man is broader than a gender, race or ethnicity.

## The hierarchy on the street

The Canadian definition of Youth Homelessness from the Canadian Observatory on Homelessness, 2016, was used by the group who ran the first national survey on youth homelessness in Canada. It states: “Youth homelessness” refers to the situation and experience of young people between the ages of 13 to 24 who are living independently of parents and/or caregivers, but do not have the means or ability to acquire a stable, safe, and consistent residence (Gaetz et al., 2016). The results of this survey will help situate the young man in my story within the context where he lives, Montreal, Quebec, Canada.

In a previous survey, published on homelessness in Canada, this group found that young people represent 20% of the homeless population in Canada (Gaetz et al., 2014). Their second survey “Without a Home: The National Youth Homelessness Survey” was done in 47 communities in 10 provinces and territories. A total of 1,103 youth experiencing homelessness were surveyed by workers in the field (Gaetz et al., 2016). The results showed that 57.9% identified as cisgender male, 36.4% as cisgender female, 1.8% as transgender, 2.5% as gender non-binary and 1.8% as two-spirit (indigenous LGBTQ2S individual), 29.5% as LGBTQ2S, 30.6% as indigenous, 28.2% as members of racialized communities, and 10.1% were from outside Canada (Gaetz et al., 2016).

It is important to remember that these are national figures that to not necessarily represent what is happening in individual cities or parts of the country. For example, it is known that some major cities, such as Toronto’, have a higher proportion of racialized youth, and the number of indigenous youths is higher in the Western provinces (Gaetz et al., 2016).

These young people as a group suffer from ongoing housing instability, high level of chronicity (ongoing homelessness for more than a year or repeated episodes over three years), nutritional vulnerability, declining mental health, low school participation, unemployment and criminal victimization (Gaetz et al., 2016). These problems are not common to all homeless youth, and many will experience intersecting forces of oppression and discrimination resulting from racism, homophobia and transphobia among others, often resulting in an inability to stay in their families and communities (Gaetz et al., 2016).

Furthermore, these vary according to individual groups, for example under the category of criminal victimization 37.4% of young women and 41.3% of transgender/gender non-binary youth reported higher levels of sexual assault in the previous 12 months (Gaetz et al., 2016). The drivers of youth homelessness, among others, were family breakdown, interpersonal violence, housing instability, mental health, and addiction issues both in the parents and/or youth themselves, and problematic transition from care, such as those in youth protection programs (Gaetz et al., 2016). Homeless young people have worsening health and well-being due to multiple factors such as inadequate nutrition, poor hygiene, lack of proper rest, high level of stress, increased risk of injury, sexual activity with multiple partners, increase exposure to STBBIs, and greater exposure to communicable diseases (Gaetz et al., 2016). The result of this includes extreme fatigue and poor mental health among other problems which leads to an inability to attend school or work, thus decreasing their chances of leaving the street (Gaetz et al., 2016). This survey provided information on homeless youth in Canada which allows me to understand who this group is, how they have come to be on the street and the problems they face once there. Therefore, I now realize that I should have spoken to the young man to better understand his issues and how I could have helped him, a white male part of the majority of homeless youth in Canada, whose health risks may not be as great as those of indigenous or racialized individuals, or females. I also do not know if he is sexually and/or gender diverse, an important risk factor for homelessness in youth.

A systematic review and meta-analysis, from Canadian researchers, also published in 2016 included studies from a number of developing countries (Embleton et al., 2016). The risks were similar with poverty being the most important risk factor globally, while in developed countries it was family conflicts (Embleton et al., 2016). Three years later another systematic review and meta-analysis of homelessness in general, from a group in Copenhagen, had similar findings including male sex, criminal behaviour and a history of incarceration, and suicide attempts as risk factors (Nilsson & Nordentoft, 2019). They stated that most of the studies were from the United States (Nilsson & Nordentoft, 2019).

Recently a systematic review, published in 2023 entitled: “Risks and resilience factors for youth homelessness in Western countries – A systematic review” echo and support the findings of the Canadian survey (Grattan et al., 2023). The risk factors they list as associated with homelessness are a history of previous homelessness and running away from home, a poor academic history, not completing high school, a history of delinquency or problem behaviour, as well as trauma particularly physical abuse, a non-heterosexual orientation, or high number of foster care placements (Grattan et al., 2023). The review reports that protective factors are not as frequently studied, but they include having a supportive and high-functioning family, higher socioeconomic status, educational attainment, and family connection. Though these were listed as protective factors, the authors did not state that they prevented homelessness (Grattan et al., 2023). They commented on ethnicity, interestingly being Hispanic was protective, but being non-white was a risk (Grattan et al., 2023). Given these studies I am more concerned about the young man from the bank, being white is potentially protective, therefore the fact that he is homeless suggests he may have had a very difficult life experience.

All these studies tell a similar story as to why young people become homeless, with significant morbidity and mortality compared to other young people (Boivin et al., 2005; Grattan et al., 2023; Milburn et al., 2024). Reviews published over the last 10 years lists some of these issues. These include a higher rate of infectious diseases such as hepatitis B, and C, HIV, mental health problems such as depression, anxiety, posttraumatic stress, attention deficit/hyperactivity disorder, bipolarity, substance use, pregnancy, high levels of violence such as physical and sexual assault, and mortality from suicide and drug overdoses (Boivin et al., 2005; Milburn et al., 2024).

## Existing resources

Several programs have been developed in different countries. Endeavours have included work done for primary prevention and to help young people who are already homeless. However, the number of interventions described in the literature is limited, and few have been adequately evaluated (Milburn et al., 2024). Gaezt et al. in their report “Without a Home” make several recommendations. One is that the breath of issues that lead to homelessness is broad and needs to be addressed at several levels of government as well as by NGOs, both nationally and internationally, and this needs to be an integrated effort (Embleton et al., 2016; Gaetz et al., 2016). They make several specific recommendations for various levels of government to assist in dealing with this issue, but they also state that more research is needed to help these various groups in developing effective programs.

Several programs related to strengthening and addressing the needs of families and youth at risk exit and are outlined in the S. O. Gaetz et al. (2016) report. One such program is “A Way Home Canada”. Their Web site will provide you with resources for school-based interventions such as addressing ADHD, bullying, family re-connection and fortifying natural supports, assistance for sexually and gender diverse youth and other minorities, employment training and education, and youth transitional programs. These various resources allow for intervention early before homelessness occurs. You may want to look at their Community Planning Toolkit. Other initiatives, also in Canada, are “Youth Reconnect”, “Link”, “Aura Host Homes”, and “The Healthy, Empowered and Resilient (HER) Pregnancy program”. A list can be found at the “Homeless Hub”. The “Geelong Project” in Australia has successfully demonstrated the effectiveness of integrated programs between the community and schools at preventing homelessness. International programs such as “Save the Children” also provide an opportunity to intervene at an early stage to prevent homelessness by assisting families and youth themselves with education, safe environments in which to live, and work, emotional support, food, and other necessities to allow them to thrive.

To assist young people who want to exit homelessness we must provide housing (Milburn et al., 2024). In the United States of America there is Permanent Supportive Housing (PSH), Housing First, and Rapid Re-Housing (RRH) which are models for older adolescents and young adults. Such models for younger adolescents are lacking so most will need to resort to foster care, if they cannot return to the home of a family member or friend (Milburn et al., 2024). The period of transition from institutional care to independent living is often a difficult period for many young people and a time when many will become homeless. Therefore, assistance in exit planning must me provided by institutions and programs for rapid housing. The previously mentioned PSH which was originally developed in Canada is such a program. This program means to “move youth out of homelessness as quickly as possible with no preconditions” (Gaetz et al., 2016) and provide them with a range of supports to reintegrate society at large. In planning such endeavours, it is important to consult young people to define what they want and to consider the culturally relevant needs of various groups of racialized and indigenous youth. Milburn in her article outlines steps for addressing research and interventions at this level (Ginsburg, 2020; Milburn et al., 2024).

## My reflections

What should I have done to help him? I should have tried to talk to him and ask what his immediate needs were? If he was hungry, my options were to give him cash or take him to a nearby restaurant and get him a meal. This would not only meet his needs but give me the opportunity to talk to him further. If he needed shelter for the night. I could help him there by reaching out to friends/colleagues who are social workers, who could give numbers I could call and try to make sure he got somewhere to stay with his dog. I could find out where to buy him warm clothes, if that was also a need. While we ate, we could chat some more and possibly find out about long term physical and mental health issues that needed to be addressed. I could suggest a variety of youth services that offer care, including the clinic where I work. There I could help him register, have the medical team doing urgent care that evening care for his acute health needs. Once he was registered his long-term medical issues could be addressed. He would also have access to an outreach worker who could also find out about previous people of importance in his life that he would like to contact. Reestablishing such contacts may lead a youth to exit homelessness (Gaetz et al., 2016; Le & Rew, 2023).

Unfortunately, I did not do that, and as I sat in my warm cab I started reflecting on my own experience in life. As a white gay adolescent/young adult living in a small community, raised in a conservative religious, low socioeconomic class family, I too, in some ways ran away from home and my community. I ran to larger and larger anonymous cities, Ottawa, Montreal, New York, where people would not know who I was and that I was a homosexual. I didn’t accept it and believed that everyone would reject me. It was easier to be anonymous. As a student, despite scholarships and grants, I could not afford much. I lived in a rooming house with older men who were alcoholics. Yet, my continued, though strained connection with my parents, and their willingness to provide me with small amounts of money, and my own educational goals, protected me from homelessness. Moreover, later I was lucky to meet a man, who became my husband, who encouraged me to be who I was and feel comfortable with myself. As a result, I am a physician, I have a home, people who love me and respect me and I am successful in my career. Giving back would not have been difficult.

## How to give back

We must become advocates for these disenfranchised and homeless young people. We can encourage different institutions in the community and government to establish processes to prevent homelessness for youth as suggested in several papers (Doukrou & Segal, 2018; Gaetz et al., 2016; Le & Rew, 2023; Milburn et al., 2024). Educating health care providers, including medical students, residents and practicing physicians to take appropriate (Doukrou & Segal, 2018) history in their offices using HEEADSSS or SHADESS to identify youth at risk and make sure that they are provided with the support and resources to avoid deterioration and future homelessness (Blukuth & Ballet, 2015; Gaetz et al., 2016; Ginsburg, 2020; Le & Rew, 2023; Milburn et al., 2024 ). With this goal in mind, I insisted that a chapter on Disenfranchised Youth be included in a book on Adolescent Health for WONCA, the World Family Medicine Association, that we hope will be published in early 2016.

It can also mean encouraging workers to intervene at the level of the family, school, or if they are in foster care assuring proper transition to independent living is guaranteed (Blukuth & Ballet, 2015; Gaetz et al., 2016; Le & Rew, 2023 ). However, if the services are not available in my community, this is where working as an advocate I could try to encourage government offices in public health and education to not only provide resources to implement appropriate programs as already exist in other provinces or countries (Gaetz et al., 2016; Grattan et al., 2023). I could also encourage or even offer continuing professional development to any adult who may be in a position to intervene with young people at risk. For youth already homeless advocating for inclusive and non-discriminatory services that help youth of various ethnic and racial background, and which are open to sexual and diverse people, appropriately gendering them, and making sure that they are protected from harassment and assault (Gaetz et al., 2016; Le & Rew, 2023; Milburn et al., 2024). Nongovernmental organizations (NGOs) often provide broad services where these young people are found. These need to be supported by the public and governments (Blukuth & Ballet, 2015; Gaetz et al., 2016). It is important to remember that a valuable source of information are the young people themselves, they know what they need. We must advocate for the inclusion of young people of the street in all levels where planning and program development is occurring (Gaetz et al., 2016). As previously mentioned, the needs vary according to the community where you live, hence informing yourself about existing resources and then using evidence-based data to supplement what is being done is important (4).

## A summary

To summarize I will borrow and add to the recommendations made by the Canadian research group in their report (Gaetz et al., 2016).

a) Intervene before youth become homeless.
b) Once the young person leaves home early interventions are key to preventing chronicity and poor health outcomes.
c) Culturally appropriate mental health support that addresses systemic discrimination for indigenous, racialized, immigrant, asylum seeking, and where appropriate conflict involved youth, which uses trauma informed methods is of utmost importance.
d) Develop interventions that are tailored to meet the needs of sexually and gender diverse youth, including interacting with community groups that deal with this population.
e) Implement services that address the specific needs of female youth.
f) Advocate at the federal, state or provincial, municipal and immediate community levels for the development of evidence-based programs and the support of NGOs who offer services that are easily accessible and aimed at this population.
g) Consult and involve homeless youth at all levels of this process, offering compensation for their expertise and time.

Unfortunately, from what I have observed and read I have learned that the street is diverse and frequently unequitable in the harm it can cause the individual. This young man piqued my interest and allowed me to define youth homelessness and understand that the street is inclusive of everyone, potentially even myself. It does not discriminate in making life difficult for anyone who becomes homeless. A hierarchy exists on the street, and it is harder for some, namely women, sexually and gender diverse groups, and depending on the community those of different ethnic and racial groups, for example indigenous youth in the west of Canada, black and other racial groups in American cities (Gaetz et al., 2016; Grattan et al., 2023). These are obvious examples, but other categories of individuals exist so be vigilant and attempt to identify who they are and help develop programs that are specific to their needs. Diversity on the street may be seen anywhere in the world. These young people may be washing your car, selling product at a market, asleep on a park bench, or huddled in a bank lobby with a dog. Therefore, when you see a young person in such a situation, don’t get in a warm taxi, as I did, speak to them, be supportive and helpful, offer them assistance and don’t harass them or abuse them verbally or worse call the authorities to intervene or give then a fine they can ill afford to pay. Remember a supportive person, a kind word, a bit of money at the right time may result in a young person exiting the street.

On a personal note, while on a tour during a recent holiday, I saw a young man asking for money at the entrance of a church. I wandered over, I could not speak to him as I did not know his language, but I took a few bills from my pocket and gave them to him. Some people in the tour saw me do this, so they did the same and the guide came over and asked him if he needed anything else. He said no, thanked us and asked if he could shake my hand. I agreed. We looked at each other and smiled. A bond had been established. What ensued I will not know, but I got back on the bus feeling, this time I have helped.
